# Fenótipo clínico da obesidade abdominal e dinapenia: *Estudo Longitudinal da Saúde dos Idosos Brasileiros* (ELSI-Brasil)

**DOI:** 10.1590/0102-311XPT233323

**Published:** 2025-01-27

**Authors:** Tatiane Melo de Oliveira, Pricilla de Almeida Moreira, Marília Santos dos Anjos, Daniela de Assumpção, Ligiana Pires Corona

**Affiliations:** 1 Universidade Estadual de Campinas, Campinas, Brasil.; 2 University of Cambridge School of Clinical Medicine, Cambridge, U.K.; 3 Universidade Estadual de Santa Cruz, Ilhéus, Brasil.

**Keywords:** Obesidade Abdominal, Idoso, Gordura Intra-Abdominal, Obesidade, Abdominal Obesity, Aged, Visceral Fat, Obesity, Obesidad Abdominal, Anciano, Grasa Intraabdominal, Obesidad

## Abstract

O objetivo do estudo é examinar a prevalência do fenótipo da obesidade abdominal dinapênica, identificado pela presença de obesidade abdominal e dinapenia, e conhecer seus fatores associados em uma amostra representativa da população brasileira. Foram usados dados da linha do base *Estudo Longitudinal da Saúde dos Idosos Brasileiros* (ELSI-Brasil) 2015-2016. A obesidade abdominal foi determinada pela razão cintura-estatura ≥ 0,55cm, e dinapenia foi identificada pela presença de baixa força de preensão palmar, por meio da dinamometria, segundo pontos de corte propostos para brasileiros. A variável dependente foi a coexistência de ambas as condições (obesidade abdominal e dinapenia), e analisou-se sua associação entre as variáveis independentes (características sociodemográficas, comportamento e condições de saúde, doenças crônicas e local de moradia segundo regiões do Brasil), utilizando-se regressão de Poisson para obter razões de prevalência brutas e ajustadas por sexo, idade e escolaridade. A prevalência de obesidade abdominal foi de 57,8%, 5,7% de dinapenia isolada e 12,3% de obesidade abdominal-dinapênica. No modelo ajustado, foram significativas as associações com tabagismo (0,7; IC95%: 0,5-0,9), consumo de álcool (0,7; IC95%: 0,5-0,9), prática de atividade física (0,6; IC95%: 0,5-0,8), autoavaliação da saúde ruim (1,7; IC95%: 1,4-2,2), multimorbidade (1,3; IC95%: 1,1-1,6), e regiões de residência. Esses fatores indicam pontos-chave para o desenvolvimento de estratégias de prevenção e tratamento da obesidade abdominal associada à baixa força muscular, e sugere-se que as metodologias aqui abordadas para seu diagnóstico sejam usadas como forma de identificação dessa condição em pessoas idosas, por sua confiabilidade e praticidade.

## Abstract (en)

This study aims to examine the prevalence of abdominal obesity-dynapenia phenotype, identified by the presence of abdominal obesity and dynapenia, and understand its associated factors with a representative sample of the Brazilian population. Data were collected from the baseline of the *Brazilian Longitudinal Study of Aging* (ELSI-Brasil) 2015-2016. Abdominal obesity was determined by a waist-to-height ratio ≥ 0.55cm, while dynapenia was identified by evidence of low handgrip strength assessed via dynamometry, according to cutoff points proposed for the Brazilian population. The dependent variable was the coexistence of both conditions (abdominal obesity and dynapenia), and its association with independent variables (sociodemographic characteristics, behavior and health conditions, chronic diseases, and place of residence by Brazilian region) was analyzed using Poisson regression to obtain crude and adjusted prevalence ratios by sex, age, and education level. The prevalence of isolated abdominal obesity was 57.8%, isolated dynapenia was 5.7%, and abdominal obesity-dynapenia was 12.3%. In the adjusted model, significant associations were found with smoking (0.7; 95%CI: 0.5-0.9), alcohol consumption (0.7; 95%CI: 0.5-0.9), physical activity (0.6; 95%CI: 0.5-0.8), poor self-rated health (1.7; 95%CI: 1.4-2.2), multimorbidity (1.3; 95%CI: 1.1-1.6), and regions of residence. These factors indicate key points for the development of prevention and treatment strategies for abdominal obesity associated with low muscle strength, and we suggest that methodologies discussed here for abdominal obesity diagnosis be used as a reliable and practical means to identify this condition in older adults.

## Abstract (es)

El objetivo del estudio es examinar la prevalencia del fenotipo de obesidad abdominal dinapénica, identificado por la presencia de obesidad abdominal y dinapenia, y comprender sus factores asociados en una muestra representativa de la población brasileña. Se utilizaron datos de la línea de base del *Estudio Longitudinal de Salud de los Ancianos Brasileños* (ELSI-Brasil) 2015-2016. La obesidad abdominal se determinó por la relación cintura-estatura ≥ 0,55cm y la dinapenia se identificó por la presencia de baja fuerza de prensión manual, mediante dinamometría, según puntos de corte propuestos para los brasileños. La variable dependiente fue la coexistencia de ambas condiciones (obesidad abdominal y dinapenia), y se analizó su asociación entre variables independientes (características sociodemográficas, comportamiento y estado de salud, enfermedades crónicas y lugar de residencia según regiones de Brasil), mediante regresión de Poisson para tasas de prevalencia crudas y ajustadas por sexo, edad y educación. La prevalencia de obesidad abdominal aislada fue del 57,8%, la de dinapenia aislada del 5,7% y la de obesidad abdominal dinapénica del 12,3%. En el modelo ajustado, se observaron asociaciones significativas con el tabaquismo (0,7; IC95%: 0,5-0,9), el consumo de alcohol (0,7; IC95%: 0,5-0,9), la actividad física (0,6; IC95%: 0,5-0,8) y la mala autoevaluación de la salud (1,7; IC95%: 1,4-2,2), multimorbilidad (1,3; IC95%: 1,1-1,6) y regiones de residencia. Estos factores indican puntos claves para el desarrollo de estrategias de prevención y tratamiento de la obesidad abdominal asociada a baja fuerza muscular, y se sugiere que las metodologías aquí abordadas para su diagnóstico se utilicen como una forma de identificar esta condición en personas mayores, debido a su confiabilidad y practicidad.

## Introdução

A população idosa do Brasil aumentou expressivamente em 56% no período de 2010 a 2022 ^1^. Paralelamente a essa transição epidemiológica, observa-se o aumento de pessoas idosas com obesidade ^2^, doença considerada a epidemia mais grave do mundo ^2^ ^,^ ^3^ ^,^ ^4^. Pesquisas representativas da população de idosos brasileiros evidenciaram prevalências de 38,8% ^5^ e 44,8% ^6^ de indivíduos com obesidade abdominal, ambas considerando a avaliação da circunferência da cintura (CC) elevada. Segundo dados da *Pesquisa Nacional de Saúde* (PNS), 24,8% dos indivíduos idosos apresentaram obesidade (índice de massa corporal - IMC > 30kg/m²) ^7^.

Durante a senescência, a reserva adiposa aumenta expressivamente, com maior acúmulo na região central ^8^ ^,^ ^9^. Além disso, alterações posturais decorrentes da senilidade óssea refletem em aumento da CC ^3^ ^,^ ^10^. Apesar disso, estudos que analisam a prevalência de obesidade abdominal em pessoas idosas brasileiras ainda são escassos ^5^ ^,^ ^6^ ^,^ ^11^.

Concomitantemente às alterações na adiposidade corporal e na concentração abdominal de gordura, ocorre também a redução da massa muscular, o aumento do infiltrado de gordura entre as fibras musculares e a redução da força muscular (dinapenia) e do desempenho físico ^12^ ^,^ ^13^. A dinapenia está associada com desfechos negativos na funcionalidade em pessoas idosas, aumentando a incapacidade, prolongando hospitalização ^14^ ^,^ ^15^ e elevando a mortalidade precoce ^6^. Dados atuais evidenciam que um quinto da população idosa brasileira apresenta dinapenia, sendo 23,7% em homens e 23,9% em mulheres ^16^.

A simultaneidade da obesidade abdominal e dinapenia dá origem ao fenótipo clínico descrito como obesidade abdominal dinapênica. Nesse fenótipo, a adiposidade central e baixa força agem em sinergia, gerando uma cascata de complicações endócrino-inflamatórias, o que pode se manifestar no idoso como hipomobilidade e/ou dependência física, podendo causar redução da funcionalidade muscular, hospitalização prolongada e morbimortalidade ^5^ ^,^ ^8^ ^,^ ^17^ ^,^ ^18^.

Estudos que apresentem a prevalência de obesidade abdominal dinapênica em indivíduos idosos brasileiros ainda são escassos ^5^ ^,^ ^11^. Considerando uma amostra de base populacional brasileira, 42,7% das pessoas idosas tinham obesidade abdominal dinapênica ^5^, usando a CC, associado à baixa força muscular com pontos de corte ≤ 26kg e ≤ 16kg para homens e mulheres, respectivamente ^19^.

Até o momento, nenhum trabalho apresentou dados de prevalência de obesidade abdominal dinapênica em idosos, considerando a razão cintura-estatura (RCE) como indicador da obesidade abdominal e pontos de cortes para baixa força muscular, específicos para a população analisada. Sendo assim, o objetivo deste estudo é examinar a prevalência do fenótipo da obesidade abdominal dinapênica em relação à presença de ambas as condições isoladas, usando ferramentas reprodutíveis na prática clínica, com pontos de corte específicos para pessoas idosas, e conhecer seus fatores associados em uma amostra representativa da população brasileira.

## Metodologia

### População do estudo

Trata-se de estudo transversal de base domiciliar que utilizou dados da linha de base do *Estudo Longitudinal da Saúde dos Idosos Brasileiros* (ELSI-Brasil), realizado entre 2015 e 2016. Ao todo, foram recrutados 9.412 participantes com idade ≥ 50 anos, residentes em 70 municípios de todas as grandes regiões do Brasil. Neste estudo, foram selecionadas informações de indivíduos com idade igual ou superior a 60 anos (n = 5.432) na ocasião da entrevista. Entre esses, 4.951 indivíduos apresentavam todas as variáveis necessárias para a composição dos fenótipos clínicos analisados e, portanto, atenderam a todos os critérios para a realização das análises.

A amostragem empregou estratificação geográfica e por conglomerados. Em municípios com até 750 mil habitantes, a amostragem foi feita em três estágios (município, setor censitário e domicílio). Em municípios maiores, em dois estágios (setor censitário e domicílio). Mais detalhes sobre o processo amostral foram publicados por Lima-Costa et al. ^20^ e estão disponíveis na página eletrônica da pesquisa (http://elsi.cpqrr.fiocruz.br).

### Variável dependente

A obesidade abdominal dinapênica foi definida pela simultaneidade dos fenótipos clínicos: obesidade abdominal e dinapenia. As variáveis que identificam esses fenótipos foram coletadas por pesquisadores previamente treinados e padronizados.

A obesidade abdominal foi identificada pela medida da RCE, que representa o resultado do valor da CC dividido pela altura ao quadrado. A CC foi aferida com fita métrica inelástica da marca Seca, no ponto médio entre a 10ª costela e a borda da crista ilíaca. O participante foi posicionado em pé, com os pés afastados e sem camisa ou blusa ^21^ ^,^ ^22^. A altura foi medida com estadiômetro vertical portátil da marca Nutri-Vida, com o participante em pé, sem sapatos e com a cabeça posicionada no plano de Frankfurt ^21^. Apresentou obesidade abdominal a pessoa idosa com valores de RCE ≥ 0,55 ^23^.

A dinapenia foi identificada pela baixa força de preensão manual, utilizando dinamômetro de preensão manual hidráulico, hidráulico (modelo SH5001, SAEHAN Corporation; http://www.saehanmedical.com/). A coleta foi feita com o participante na posição sentada, segurando o dinamômetro com a mão dominante e com os braços junto ao corpo, com o cotovelo dominante, formando um ângulo de 90º. Foram orientados a apertar o dispositivo com a mão dominante o mais forte possível por dois segundos. Antes da aplicação do teste definitivo, foi realizado um teste de familiarização com a mão não dominante ^15^ ^,^ ^21^. O teste foi então realizado três vezes no membro dominante, com um minuto de descanso entre os testes, e o maior valor entre as três tentativas foi escolhido para as análises. Foram classificados como dinapênicos os indivíduos idosos com força inferior ao percentil 20, segundo faixa etária e sexo, com base nos pontos de corte propostos por Moreira et al. ^17^, baseados na análise de dados do próprio ELSI-Brasil. Os indivíduos foram considerados dinapênicos com base nos seguintes valores de força de preensão manual, medidos em quilogramas-força (kg/F): para mulheres idosas, < 16kg/F para idades entre 60-69 anos, < 14kg/F para idades entre 70-74 anos, < 13kg/F para idades entre 75-79 anos, < 12kg/F para idades entre 80-84 anos, < 10kg/F para idades entre 85-110 anos. Para homens idosos, os valores considerados foram: < 27kg/F para idades entre 60-64 anos, < 26kg/F para idades entre 65-69 anos, < 24kg/F para idades entre 70-74 anos, < 22kg/F para idades entre 75-79 anos, < 19kg/F para idades entre 80-84 anos, < 16kg/F para idades entre 85-110 anos ^17^. Considerando que, durante a senescência, é esperada uma perda de força muscular inerente ao envelhecimento biológico, optou-se por usar pontos de corte estabelecidos especificamente para a população idosa de comunidade. Isso se deve ao fato de que pontos de referências baseados em populações adultas e saudáveis poderia aumentar artificialmente a prevalência da dinapenia. Dada a representatividade nacional da amostra de pessoas idosas de comunidade, considerou-se o método proposto por Moreira et al. ^17^ como o mais adequado para classificar baixa força muscular em pessoas idosas no Brasil.

### Variáveis independentes

As covariáveis selecionadas neste estudo foram coletadas com base em questionário padronizado e por pesquisadores treinados:

(a) Características sociodemográficas: idade (anos completos); sexo (masculino e feminino); escolaridade (anos completos de estudo); situação conjugal (com cônjuge: casado, amasiado ou união estável; sem cônjuge: solteiro, divorciado, separado ou viúvo); cor de pele autorreferida (branca e não branca: preta; parda; amarela; indígena). Para fins de análise, a variável cor de pele foi dicotomizada em branca e não branca. Também foi incluída a posse de plano médico de saúde (sim/não).

(b) Comportamentos de saúde: tabagismo (nunca fumou; fumou menos de 100 cigarros na vida; ex-fumante - fumou mais de 100 cigarros na vida, mas havia parado de fumar no momento da entrevista; fumantes atuais); consumo regular de álcool (ingere uma dose ou mais, uma vez por mês ou mais); autoavaliação de saúde (muito boa, regular, ruim/muito ruim); e prática de atividade física insuficiente avaliada por meio da versão abreviada do *Questionário Internacional de Atividade Física* (IPAQ), validada para o Brasil ^24^, sendo classificados como insuficientemente ativos os participantes que realizavam menos de 150 minutos de atividade física por semana ^25^.

(c) Condições de saúde: multimorbidade definida pela presença autorreferida de duas ou mais doenças crônicas não transmissíveis (DCNT) ^26^ (nenhuma, uma ou duas e mais DCNT). As seguintes morbidades foram selecionadas: hipertensão arterial sistêmica (HAS); diabetes mellitus, doenças cardíacas, artrite, osteoporose, acidente vascular encefálico. A ausência de informações sobre a doença foram excluídas da análise.

(d) Regiões do Brasil de moradia dos idosos: Norte, Nordeste, Sudeste, Sul e Centro-oeste.

### Análise estatística

Os dados descritivos foram expressos como frequências absolutas e relativas, medidas de tendência central (média ou mediana) e dispersão (desvio-padrão [DP] ou intervalo interquartílico) de acordo com a normalidade dos dados. Testou-se a normalidade dos dados por meio de histogramas e do teste de Kolmogorov-Smirnov. A comparação de variáveis categóricas foi realizada por meio do teste qui-quadrado de Pearson. Já os testes de Mann-Whitney, t de Student e Kruskal-Wallis foram utilizados para verificar a diferença entre as variáveis numéricas, de acordo com a respectiva normalidade.

As associações entre ter o fenótipo obesidade abdominal dinapênica e as variáveis independentes foram verificadas por meio de regressão de Poisson, que estimou razões de prevalência brutas (RP bruta) e ajustadas (RP ajustada) por sexo, idade e escolaridade. A escolha das variáveis para ajustes foi teórica, considerando a associação significativa com a variável de desfecho (obesidade abdominal dinapênica) e por serem determinantes clássicos de saúde utilizados como fatores de confusão na maioria dos estudos epidemiológicos. Considerou-se significativos valores de p < 0,05.

As análises foram realizadas por meio do programa Stata 14.0 (https://www.stata.com), utilizando-se o módulo *survey*, que permite considerar a estrutura complexa da amostra com a atribuição de pesos amostrais.

### Aspectos éticos

O ELSI-Brasil foi aprovado pelo Comitê de Ética e Pesquisa do Instituto René Rachou da Fundação Oswaldo Cruz, Minas Gerais (CAAE 34649814.3.0000.5091), e todos os participantes assinaram o Termo de Consentimento Livre e Esclarecido antes das entrevistas e das avaliações físicas.

## Resultados

Dos 4.951 indivíduos selecionados, 54,8% eram mulheres, 58,1% apresentavam idade entre 60-69 anos, 59,8% viviam com o cônjuge, 44,2% tinham apenas quatro anos de estudo e 54,9% se autodeclararam como não brancos. As prevalências de foram de 57,8% para obesidade abdominal isolada, 5,7% de dinapenia isolada e 12,3% para obesidade abdominal dinapênica (Tabela 1).

**Tabela 1:** Prevalência de obesidade abdominal, dinapenia e obesidade abdominal dinapênica, segundo características sociodemográficas de pessoas idosas de comunidade. *Estudo Longitudinal da Saúde dos Idosos Brasileiros* (ELSI-Brasil), 2015-2016.

Variáveis	n (%)	Sem obesidade abdominal ou dinapenia	Obesidade abdominal	Dinapenia	Obesidade abdominal dinapênica	Valor de p *
		% (IC95%)	% (IC95%)	% (IC95%)	% (IC95%)	
Amostra total	4.951 (100,0)	24,2 (22,5-25,9)	57,8 (55,3-60,1)	5,7 (4,7-7,0)	12,3 (10,7-14,0)	
Sexo									< 0,001
Feminino	2.926 (54,8)	20,1 (19,7-23,0)	59,6 (55,9-63,2)	4,9 (3,7-6,6)	14,4 (12,3-16,8)	
Masculino	2.025 (45,2)	28,1 (25,5-30,8)	55,5 (52,9-58,1)	6,6 (5,2-8,4)	9,7 (8,0-11,9)	
Faixa etária (anos)						0,005
60-69	2.704 (58,1)	26,2 (24,1-28,5)	55,2 (52,5-57,8)	6,3 (5,0-7,8)	12,2 (10,4-14,3)	
70-79	1.618 (29,9)	21,6 (19,3-24,1)	61,8 (58,7-64,9)	5,0 (3,6-6,9)	11,6 (9,4-14,2)	
≥ 80	629 (12,0)	20.7 (17,1-24,8)	60,6 (54,7-66,1)	4,7 (3,0-7,2)	14,1 (11,1-17,7)	
Situação conjugal						0,005
Com cônjuge	2.627 (59,8)	23,9 (22,0-26,0)	59,9 (57,4-62,4)	4,9 (3,9-6,1)	11,2 (9,5-13,2)	
Sem cônjuge	2.324 (40,2)	24,6 (22.0-27.3)	54,7 (51,1-58,2)	6,9 (5,3-8,9)	13,8 (11,9-15,9)	
Escolaridade (anos)						0,002
0	1.061 (17,9)	21,2 (18,6-24,0)	54,4 (48,3-60,4)	7,8 (5,5-11,0)	16,6 (13,5-20,1)	
1-4	2.158 (44,2)	22,9 (20,2-25,8)	59,3 (56,3-62,2)	5,9 (4,5-7,7)	11,9 (10,1-14,0)	
5-8	807 (17,2)	26,0 (22,9-29,3)	57,9 (53,7-61,9)	4,5 (3,05-6,7)	11,6 (8,7-15,3)	
≥ 9	925 (20,6)	28,0 (24,9-31,7)	57,7 (53,9-61,3)	4,4 (2,9-6,8)	9,8 (7,8-12,3)	
Raça (autodeclarada)						0,014
Branca	1.974 (45,1)	24,4 (22,1-26,9)	60,0 (57,2-62,8)	4,6 (3,7-5,8)	10,9 (9,2-12,9)	
Não branca	2.765 (54,9)	24,3 (21,9-26,8)	55,7 (52,7-58,7)	6,5 (5,1-8,3)	13,4 (11,3-15,8)	
Posse de plano de saúde						0,031
Não	3.690 (73,3)	24,1 (22,3-26,0)	56,6 (53,5-59,6)	6,1 (4,8-7,8)	13,2 (11,3-15,3)	
Sim	1.255 (26,7)	24,1 (21,3-27,2)	61,5 (58,5-64,5)	4,7 (3,2-6,8)	9,7 (7,8-12,0)	
Região do Brasil **						< 0,001
Norte	376 (5,4)	19,9 (15,5-25,1)	57,2 (52,2-62,1)	5,3 (3,6-7,7)	17,5 (12,8-23,4)	
Nordeste	1.314 (23,3)	21,2 (18,4-24,2)	50,7 (44.5-57,0)	8,8 (6,2-12,5)	19,3 (15,3-23,9)	
Sudeste	2.057 (47,8)	26,8 (24,3-29,5)	59,1 (56,8-61,4)	4,4 (3,2-6,0)	9,6 (7,9-11,7)	
Sul	701 (17,2)	23,0 (20,0-26,4)	63,2 (57,7-68,4)	6,4 (3,2-12,7)	8,9 (6,5-11,9)	
Centro-oeste	503 (6,4)	22,5 (19,7-25,6)	60,1 (54,5-65,4)	6,8 (3,6-12,5)	10,9 (8,3-14,2)	

IC95%: intervalo de 95% de confiança.

* Mann Whitney, t de Student e Kruskal-Wallis;

** Região de moradia de pessoas idosas.

A prevalência de obesidade abdominal dinapênica foi significativamente maior no sexo feminino (14,4%), nos longevos (14,1%), nos que viviam sem cônjuge (13,8%), que nunca estudaram (16,6%), que se autodeclararam não brancos (13,4%) e nos que não possuíam plano de saúde (13,2%). Em relação às regiões do país, a maior prevalência de obesidade abdominal isolada foi registrada na Região Sul (63,2%). Na Região Nordeste, houve maior prevalência de obesidade abdominal dinapênica (19,3%) e de dinapenia isolada (8,8%) (Tabela 1).

A Tabela 2 apresenta as prevalências segundo os comportamentos de saúde e a presença de multimorbidade. Entre os indivíduos idosos fumantes, observou-se menor prevalência de obesidade abdominal isolada (39,8%) e obesidade abdominal dinapênica (9%) e maior prevalência de dinapenia isolada (8,9%), comparado aos outros grupos. O consumo de álcool foi associado à menor prevalência de todos os fenótipos (obesidade abdominal: 56,9%, dinapenia: 4,2%, obesidade abdominal dinapênica: 8,6%) comparado ao grupo que não fazia ingestão de álcool. Ser fisicamente ativo foi associado à menor prevalência de dinapenia isolada (5,1%) e obesidade abdominal dinapênica (10%). Pessoas idosas com duas ou mais morbidades, tiveram maior prevalência de obesidade abdominal isolada (61,4%) e de obesidade abdominal dinapênica (14,2%).

**Tabela 2:** Prevalência de obesidade abdominal, dinapenia e obesidade abdominal dinapênica segundo comportamentos em saúde e multimorbidade em pessoas idosas de comunidade. *Estudo Longitudinal da Saúde dos Idosos Brasileiros* (ELSI-Brasil), 2015-2016.

Variáveis	n (%)	Sem obesidade abdominal ou dinapenia	Obesidade abdominal	Dinapenia	Obesidade abdominal dinapênica	Valor de p *
		% (IC95%)	% (IC95%)	% (IC95%)	% (IC95%)	
Tabagismo						< 0,001
Nunca fumou	2.252 (45,5)	20,8 (18,8-23,1)	60,6 (57,6-63,5)	4,9 (3,9-6,3)	13,6 (11,7-15,8)	
Ex-fumante	1.985 (40,1)	21,6 (19,2-24,2)	61,2 (58,0-64,2)	5,5 (4,2-7,2)	11,8 (9,9-13,9)	
Fumante	711 (14,3)	42,2 (37,5-47,0)	39,8 (35,0-44,7)	8,9 (6,1-12,8)	9,0 (6,9-12,1)	
Consumo de álcool						< 0,001
Não ingere	3.861 (75,3)	22,1 (20,3-23,9)	58,2 (55,3-61,1)	6,2 (5,1-7,6)	13,5 (11,7-15,5)	
Ingere (≥ 1) **	1.086 (24,7)	30,2 (27,5-33,1)	56,9 (53,7-60,0)	4,2 (2,7-6,4)	8,6 (6,8-10,9)	
Multimorbidade (DCNT)						< 0,001
Nenhuma ou 1	2.307 (49,1)	28,8 (26,6-31,1)	54,2 (50,9-57,4)	6,9 (5,2-9,2)	10,1 (8,3-12,1)	
2 ou mais	2.472 (50,9)	19,6 (17,4-22,0)	61,4 (58,2-64,5)	4,7 (3,7-6,0)	14,2 (12,3-16,3)	
Prática de atividade física						< 0,001
Insuficientemente ativos ***	1.819 (35,9)	21,3 (18,6-24,2)	55,7 (51,0-60,3)	6,7 (5,0-9,0)	16,3 (13,8-19,1)	
Fisicamente ativos	3.132 (64,1)	25,8 (24,0-27,7)	59,0 (57,0-61,0)	5,1 (4,1-6,4)	10,0 (8,5-11,7)	
Autoavaliação de saúde						< 0,001
Muito bom	2.060 (42,9)	28,3 (26,0-30,6)	56,5 (53,3-59,6)	5,0 (3,3-7,4)	10,3 (8,6-12,2)	
Regular	2.284 (45,5)	21,9 (19,8-24,2)	60,3 (57,7-62,9)	10,0 (7,5-13,1)	12,4 (10,5-14,6)	
Ruim/Muito ruim	594 (11,5)	17,8 (14,6-21,7)	53,0 (47,9-58,1)	5,7 (4,6-7,0)	19,2 (15,5-23,5)	

DCNT: doenças crônicas não transmissíveis; IC95%: intervalo de 95% de confiança.

* Mann Whitney, t de Student e Kruskal-Wallis;

** Uma vez ou mais por mês;

*** Nível suficiente: pelo menos 150 minutos/semana, incluindo caminhada e atividades de intensidade moderada ou vigorosa.

**Figura 1:**
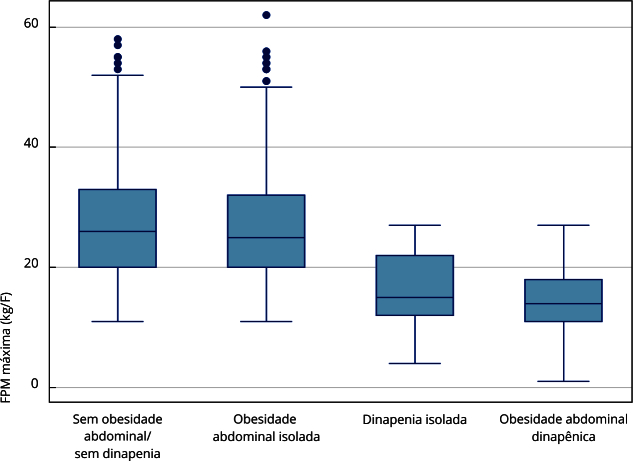
Comparação entre as variabilidades da força máxima, de acordo com os diferentes fenótipos clínicos analisados em pessoas idosas de comunidade. *Estudo Longitudinal da Saúde dos Idosos Brasileiros* (ELSI-Brasil), 2015-2016.

As Figuras 1 e 2 mostram os diagramas de caixa (*boxplot*) com a variabilidade da força máxima e da RCE de acordo com as condições clínicas analisadas. A maior mediana da força foi observada entre os idosos sem obesidade abdominal e sem dinapenia (28kg/F), e a menor no grupo com obesidade abdominal dinapênica (15kg/F; p < 0,001). A maior mediana da RCE foi observada nos grupos com obesidade abdominal e obesidade abdominal dinapênica (0,63 nos dois grupos; p < 0,001).

FPM: força de pressão palmar. Nota: teste Kruskal-Wallis; valor de p < 0,000.

**Figura 2:**
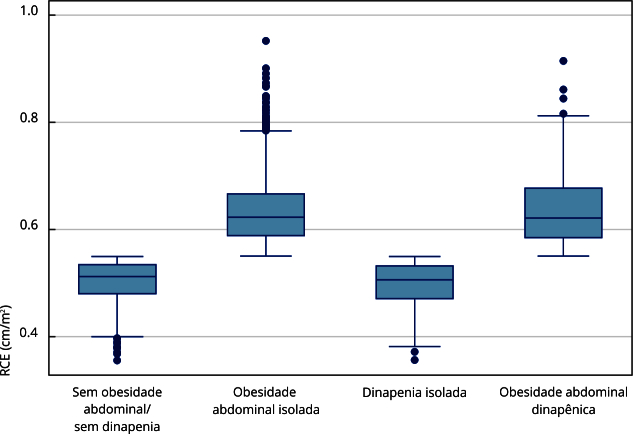
Comparação da variabilidade da razão cintura-estatura (RCE) média, de acordo com os diferentes fenótipos clínicos analisados de amostra em pessoas idosas de comunidade. *Estudo Longitudinal da Saúde dos Idosos Brasileiros* (ELSI-Brasil), 2015-2016.

Nota: teste Kruskal-Wallis; valor de p < 0,000.

Na Tabela 3, estão apresentadas as RP bruta e ajustada dos fatores associados à obesidade abdominal dinapênica. Após o modelo ajustado por sexo, idade e escolaridade, permaneceram significativas as associações inversas com tabagismo atual, consumo de álcool, prática de atividade física insuficiente e regiões de moradia das pessoas idosas no Brasil. Associações positivas foram identificadas com autoavaliação da saúde ruim ou muito ruim e presença de multimorbidade.

**Tabela 3:** Razão de prevalência (RP) bruta e ajustada de obesidade abdominal dinapênica, segundo dados descritivos da amostra de pessoas idosas de comunidade. *Estudo Longitudinal da Saúde dos Idosos Brasileiros* (ELSI-Brasil), 2015-2016.

Variáveis	RP bruta (IC95%)	Valor de p *	RP ajustada ** (IC95%)	Valor de p *
Tabagismo				
Nunca fumou	1,00		1,00	
Ex-fumante	0,8 (0,7-1,0)	0,112	0,9 (0,7-1,1)	0,406
Fumante	0,7 (0,5-0,8)	0,002	0,7 (0,5-0,9)	0,004
Consumo de álcool				
Não ingere	1,00	< 0,001	1,00	0,009
Ingere (≥ 1) ***	0,6 (0,5-0,8)		0,7 (0,5-0,9)	
Prática de atividade física				
Insuficientemente ativos ^#^	1,00		1,00	
Fisicamente ativos	0,6 (0,5-0,7)	< 0,001	0,6 (0,5-0,8)	< 0,001
Autoavaliação de saúde				
Muito bom	1,00		1,00	
Regular	1,2 (1,0-1,4)	0,041	1,2 (1,0-1,4)	0,049
Ruim/Muito ruim	1,9 (1,4-2,3)	< 0,001	1,7 (1,4-2,2)	< 0,001
Multimorbidade (DCNT)				
Nenhuma ou 1	1,00		1,00	
2 ou mais	1,5 (1,2-1,9)	< 0,001	1,3 (1,1-1,6)	< 0,001
Região do Brasil ^##^				
Norte	1,00		1,00	
Nordeste	1,1 (0,7-1,6)	0,619	1,0 (0,7-1,5)	0,798
Sudeste	0,5 (0,4-0,8)	0,002	0,5 (0,4-0,8)	< 0,001
Sul	0,5 (0,3-0,7)	0,002	0,5 (0,3-0,8)	0,002
Centro-oeste	0,6 (0,4-0,9)	0,018	0,6 (0,4-0,9)	0,015

DCNT: doenças crônicas não transmissíveis; IC95%: intervalo de 95% de confiança.

* Regressão de Poisson;

** No modelo de análise ajustada, foram selecionadas as variáveis sexo, idade e escolaridade;

*** Uma vez ou mais por mês;

^#^ Nível suficiente: pelo menos 150 minutos/semana, incluindo caminhada e atividades de intensidade moderada ou vigorosa;

^##^ Região de moradia de pessoas idosas.

## Discussão

Os resultados deste estudo representam 31,2 milhões de pessoas idosas brasileiras, constatando que uma a cada nove apresenta o fenótipo obesidade abdominal dinapênica, sendo, em sua maioria, do sexo feminino, longevas, sem vida conjugal e não alfabetizadas. Mais da metade da população idosa brasileira avaliada apresenta obesidade abdominal isolada, sendo a maioria mulheres, indivíduos mais jovens, com vida conjugal e com até quatro anos de estudo. A menor prevalência entre as condições clínicas analisadas foi a dinapenia isolada, que, diferentemente dos outros fenótipos, foi maior entre os homens, indivíduos idosos mais jovens, não alfabetizados e sem vida conjugal.

Para avaliar a obesidade abdominal na população idosa, o indicador precisa considerar as mudanças na estatura e na redistribuição da gordura, comuns no envelhecimento ^9^, além da grande variação de estatura influenciada pela diversidade étnica. Nesse contexto, entende-se que a CC isolada pode não ser o melhor indicador de obesidade abdominal na população idosa ^9^ ^,^ ^27^, pois não considera tais modificações e influências, além da ausência de pontos de cortes específicos para essa população ^27^. Pensando na identificação da gordura visceral e em corrigir as limitações da CC isolada, estudos mais recentes propõem o uso da RCE para a identificação do fenótipo da obesidade abdominal em pessoas idosas ^27^.

Apesar de não ser característico da mulher adulta ter distribuição de gordura do tipo androide ^28^, neste estudo, foi observado um número elevado de idosas brasileiras com obesidade abdominal. Isso pode ser explicado por mudanças hormonais associadas à menopausa, que impactam na composição corporal da mulher, levando à maior predisposição ao acúmulo de gordura na região abdominal ^9^. Além disso, é importante ressaltar que existe um perfil inflamatório crônico, estresse oxidativo elevado e resistência à insulina associados à distribuição de gordura do tipo androide-visceral ^3^ ^,^ ^29^.

A partir dos 70 anos, há uma tendência à redução da massa corporal total e da massa magra funcional e isso pode explicar o fato de a obesidade abdominal isolada ir diminuindo e obesidade abdominal dinapênica ir aumentando com o avanço da idade. No entanto, os indivíduos idosos que envelhecem com obesidade abdominal podem ter declínio mais acelerado da força muscular, em função dos mecanismos inflamatórios envolvidos na lipotoxicidade do tecido gorduroso visceral ^30^. É importante considerar que a força muscular diminui em uma taxa muito maior do que a massa muscular ^31^, predispondo ao desenvolvimento de dinapenia ^32^. Segundo Borges et al. ^16^, a cada ano vivido após os 60 anos, espera-se uma redução de 0,11kg na força de preensão.

Neste artigo, o grupo com maior escolaridade teve menor prevalência de todos os fenótipos, exceto da obesidade abdominal isolada, que se manteve acima de 50% em todos os níveis de escolaridade. Esses resultados corroboram com publicações que abordam que o baixo nível de escolaridade e a inatividade física estão entre os fatores de riscos modificáveis para baixa força muscular em pessoas idosas ^16^.

Neste estudo, foi observada menor prevalência de obesidade abdominal dinapênica em idosos que bebem ou fumam, e essa associação negativa se mantém significativa após ajuste. Duas hipóteses podem justificar esses resultados: (1) viés de sobrevivência: pessoas que usam tabaco e álcool tendem a morrer mais precocemente ^3^ ^,^ ^33^; (2) a nicotina pode atuar regulando funções neuroquímicas ligadas aos mecanismos de fome e saciedade. No entanto, vale ressaltar que apenas 14% das pessoas idosas avaliadas eram fumantes.

A realização de pelo menos 150 minutos de atividade física por semana reduziu a chance de ter o fenótipo obesidade abdominal dinapênica neste estudo. Esses resultados confirmam as evidências de que manter-se ativo, atingindo o tempo mínimo recomendado, protege contra a perda de força e o excesso de gordura corporal ^16^. Considerando as mudanças no estilo de vida, a prática de atividade física é proposta como uma intervenção de primeira linha para prevenir e tratar a fraqueza muscular em indivíduos idosos; no entanto, essa prática pode ser dificultada ou impedida quando há declínio na capacidade funcional. A respeito disso, é consenso na literatura que pessoas idosas com capacidade funcional prejudicada requisitam os serviços de saúde com mais frequência ^34^.

Os achados indicam que, quanto maior o número de doenças crônicas acumuladas, maior é a prevalência de obesidade abdominal e obesidade abdominal dinapênica em pessoas idosas. Em países como o Brasil, está documentado um aumento na prevalência das doenças crônicas e isso reflete no perfil de morbidade da população idosa ^29^. Além disso, o próprio envelhecimento está associado ao desenvolvimento de DCNT ^35^ ^,^ ^36^. Considerando que a obesidade é associada a mais de 230 comorbidades e complicações ^37^, se a pessoa idosa já envelhece com obesidade central, será mais um incremento negativo na sua capacidade funcional ^3^ ^,^ ^38^.

A inflamação crônica de baixo grau e sistêmica, conhecida por inflammaging, fortemente associada ao envelhecimento, é caracterizada pela maior circulação de mediadores inflamatórios. Somada a isso, a fisiopatologia das doenças crônicas como diabetes mellitus, HAS, obesidade e outras também compartilham vias de inflamação sistêmica ^3^ ^,^ ^29^. Isto é, o indivíduo com obesidade abdominal e multimorbidade enfrenta tripla carga inflamatória, a do *inflammaging*, das disfunções inflamatórias geradas pelas doenças crônicas e do excesso de adiposidade visceral.

Neste estudo, a prevalência de obesidade abdominal foi maior em residentes na Região Sul, seguido do Centro-oeste do Brasil, e a menor prevalência de obesidade abdominal na Região Nordeste, seguida da Região Norte. A maior prevalência de dinapenia e de obesidade abdominal dinapênica foi verificada na Região Nordeste do Brasil. A menor prevalência de dinapenia e de obesidade abdominal dinapênica foi observada na Região Sudeste. Costa et al. ^38^ avaliaram 1.844 indivíduos idosos da Região Sul do Brasil e encontraram 29% de pessoas idosas com obesidade pelo IMC e 50,4% com obesidade abdominal considerando a CC elevada. Em estudo conduzido na Região Centro-oeste, foram observadas 65,5% das mulheres idosas com CC elevada ^39^. Até o momento, esse é o primeiro estudo de base populacional que apresenta dados de prevalência dos fenótipos analisados em todas as regiões brasileiras de forma comparativa.

Resultado divergente sobre a prevalência de obesidade foi identificado pela *Vigilância de Fatores de Risco e Proteção para Doenças Crônicas por Inquérito Telefônico* (Vigitel) ^40^. Segundo dados coletados pelo inquérito populacional, na Região Sul, há o menor percentual de idosos com obesidade e, no Norte, o maior percentual. As diferenças encontradas podem ser explicadas pelas diferenças metodológicas para o diagnóstico de obesidade. O inquérito Vigitel usou o IMC, calculado a partir de dados de peso e altura autorrelatados, como parâmetro de identificação da obesidade, que não discrimina composição corporal ou local de concentração da adiposidade ^29^ ^,^ ^41^ e, por isso, não deve ser usado como único indicador para avaliar a obesidade ^42^ em qualquer idade, e especialmente no envelhecimento, já que pessoas idosas com obesidade abdominal podem ter IMC normal, pois nessa fase da vida a perda de massa magra é compensada pelo aumento de gordura ^3^ ^,^ ^29^.

Como limitação do estudo, há o desenho transversal, que restringe a avaliação da causalidade entre fatores associados à obesidade abdominal dinapênica. Além disso, embora a pesquisa seja baseada em instrumentos validados, variáveis autorreferidas, como a quantificação do tempo de atividade física, podem estar sujeitas a viés de recordação.

Como pontos fortes deste estudo, compete destacar que este foi o primeiro a explorar dados de prevalência do fenótipo obesidade abdominal dinapênica e das duas condições isoladas, examinando os fatores associados em uma amostra representativa de indivíduos idosos brasileiros que vivem em comunidade. Os métodos de avaliação da obesidade e da dinapenia escolhidos nesta análise levam em consideração o baixo custo, a fácil aplicação e a reprodutibilidade dos métodos na prática clínica, pensando no diagnóstico precoce e escolhendo pontos de corte ajustados que consideram as mudanças fisiopatológicas da composição corporal durante o envelhecimento.

## Conclusão

Com base nos resultados deste estudo, observou-se que metade das pessoas idosas brasileiras analisadas tem excesso de gordura abdominal. A dinapenia isolada foi menos comum, mas quando combinada com obesidade abdominal, sua prevalência mais que duplicou. Mulheres tiveram maior prevalência de obesidade com ou sem dinapenia. A obesidade abdominal foi mais comum entre os indivíduos idosos jovens, enquanto a obesidade abdominal dinapênica foi mais prevalente entre pessoas idosas longevas. Além disso, nas regiões Sul e Centro-oeste, seis em cada 10 indivíduos idosos apresentaram obesidade abdominal isolada, enquanto, no Nordeste e no Norte a prevalência de obesidade abdominal dinapênica foi maior.

Considerando todos os impactos negativos da obesidade abdominal relacionados ao envelhecimento, é necessário avaliar a inclusão de medidas que identifiquem a obesidade abdominal, além da obesidade geral, nos inquéritos populacionais.

Entre as implicações práticas dos resultados apresentados, sugere-se que as metodologias abordadas para o diagnóstico de obesidade abdominal dinapênica possam ser usadas como forma de identificação precoce ou de prevenção de progressão do fenótipo da obesidade central associado à baixa força, considerando sua confiabilidade e praticidade. Ademais, o estímulo à prática de atividade física e a prevenção das DCNT são pontos chave para reduzir essa condição. Análises como esta podem contribuir para a elaboração de estratégias que possam, de alguma forma, favorecer a redução ou o controle da obesidade abdominal dinapênica na população idosa brasileira.
